# Hydrogen Peroxide Induce Human Cytomegalovirus Replication through the Activation of p38-MAPK Signaling Pathway

**DOI:** 10.3390/v7062748

**Published:** 2015-06-04

**Authors:** Jun Xiao, Jiang Deng, Liping Lv, Qiong Kang, Ping Ma, Fan Yan, Xin Song, Bo Gao, Yanyu Zhang, Jinbo Xu

**Affiliations:** 1 Beijing Key Laboratory of Blood Safety and Supply Technologies, Beijing 100850, China; E-Mails: ammsxj@126.com (J.X.); ammsdjxm@163.com (J.D.); lvlp2007@sina.com (L.L.); kangq88@163.com (Q.K.); maping1111@hotmail.com (P.M.); yanff2004@126.com (F.Y.); xinxinsophine01@126.com (X.S.); gaobolove@sina.com (B.G.); 2 Beijing Institute of Transfusion Medicine, 27 (9) Taiping Road, Beijing 100850, China

**Keywords:** CMV, H_2_O_2_, IE1, viral replication, antioxidants, p38-MAPK

## Abstract

Human cytomegalovirus (HCMV) is a major risk factor in transplantation and AIDS patients, which induces high morbidity and mortality. These patients infected with HCMV experience an imbalance of redox homeostasis that cause accumulation of reactive oxygen species (ROS) at the cellular level. H_2_O_2_, the most common reactive oxygen species, is the main byproduct of oxidative metabolism. However, the function of H_2_O_2_ on HCMV infection is not yet fully understood and the effect and mechanism of *N*-acetylcysteine (NAC) on H_2_O_2_-stimulated HCMV replication is unclear. We, therefore, examined the effect of NAC on H_2_O_2_-induced HCMV production in human foreskin fibroblast cells. In the present study, we found that H_2_O_2_ enhanced HCMV lytic replication through promoting major immediate early (MIE) promoter activity and immediate early (IE) gene transcription. Conversely, NAC inhibited H_2_O_2_-upregulated viral IE gene expression and viral replication. The suppressive effect of NAC on CMV in an acute CMV-infected mouse model also showed a relationship between antioxidants and viral lytic replication. Intriguingly, the enhancement of HCMV replication via supplementation with H_2_O_2_ was accompanied with the activation of the p38 mitogen-activated protein kinase pathway. Similar to NAC, the p38 inhibitor SB203580 inhibited H_2_O_2_-induced p38 phosphorylation and HCMV upregulation, while upregulation of inducible ROS was unaffected. These results directly relate HCMV replication to H_2_O_2_, suggesting that treatment with antioxidants may be an attractive preventive and therapeutic strategy for HCMV.

## 1. Introduction

Human cytomegalovirus (HCMV), a β herpesvirus, is an enveloped, large and double-stranded DNA virus. Like most herpesviruses, HCMV is able to establish a latent state in its hosts after primary infection, which can result in serious health conditions when the virus reactivates and performs lytic replication [1]. HMCV infection can be asymptomatic among immunocompetent people, but it can become an important and common cause of morbidity and mortality in immunocompromised patients, such as those with AIDS, solid organ transplantation, and hematopoietic stem cell transplantation [1,2,3,4,5]. Previous studies have shown that HCMV infection is highly associated with atherosclerosis, cardiovascular diseases [6,7,8], and inflammatory bowel disease [9].

Several mechanisms of the regulation of HCMV and mouse cytomegalovirus (MCMV) latency, reactivation, and lytic replication, such as chromatin remodeling and mitogen-activated protein kinase (MAPK) pathways, have been reported by previous studies [10,11,12,13,14]. However, none of these proposed triggers can be the cause of all clinical cases of HCMV reactivation and replication.

Although the common physiological trigger that stimulates HCMV replication remains unclear, many clinical diseases are characterized by high levels of oxidative stress. Patients who undergo solid organ transplantation generally suffer from oxidative stress and inflammation associated with ischemia/reperfusion, organ rejection, and as a side effect of immunosuppressive therapy [15,16,17]. AIDS patients have high levels of oxidative stress and inflammation as a result of the defensive mechanism of the immune system in the response to HIV infection [18]. In cardiovascular diseases, oxidative stress and inflammation can also be found and are believed to contribute to the development of atherosclerosis [19,20]. Thus, one potential key to determining the trigger of HCMV replication may be oxidative stress.

As a byproduct of oxidative metabolism [21], H_2_O_2_ was produced and released to impair redox homeostasis during oxidative stress. Mechanically, transcription of the major HCMV immediate early (IE) gene is driven by the complex major IE (MIE) promoter/enhancer. Within the region, there are several binding sites for known cellular transcription factors [22], such as NF-κB, CREB, ATF, and YY1. Studies have shown that high levels of oxidative stress result in activation of these transcription factors through mitogen- and stress-activated protein kinase (MSK), which is activated by the p38-MAPK pathway [23,24].

Thus, we hypothesize that the activation of p38-MAPK signaling plays a significant role in the HCMV replication caused by hydrogen peroxide, and this could be decreased by antioxidant treatment.

## 2. Materials and Methods

### 2.1. Cell Culture, Chemical Reagents and Antibodies

Human foreskin fibroblast (HFF) cells of no more than 15 passages, HEK 293 cells, mouse embryonic fibroblast (MEF) cells, and MRC-5 cells were cultured in Dulbecco’s modified Eagle’s Medium (DMEM) supplemented with 10% fetal bovine serum (FBS) at 37 °C under a 5% CO_2_ atmosphere. Confluent cell monolayers were starved from serum for 24 h before infection. Serum free DMEM was used during drug treatment, virus incubation, and infection until the cells were harvested.

H_2_O_2_ solution, 3-amino-1,2,4-triazole (ATA), *N*-acetylcysteine (NAC), bovine liver catalase, reduced l-glutathione, 2′,7′-dichlorodihydrofluorescein diacetate (H_2_DCF-DA) and the p38 inhibitor SB203580 were purchased from Sigma Life Science (St. Louis, MO, USA).

The rabbit polyclonal antibodies used in this study included phospho-p38 (T180/Y182), p38 and β-actin (all from ABclonal technology, Cambridge, MA, USA) and the mouse monoclonal antibodies to HCMV, pp72 and pp65, were purchased from Santa Cruz Biotechnology (Santa Cruz, CA, USA).

### 2.2. Plasmids

The MIEP-pGL3 and pRL-TK plasmids were kindly provided by Dr. Dongqing Wen [25]. The plasmid expression specific siRNA used to target human catalase (5′-GCCTGGGACCCAATTATCTTCATAT-3′) and the scrambled control siRNA were purchased from Hanbio (Shanghai, China). The transfection of HEK 293 and HFF cells was performed using Lipofectamine 3000 reagent from Invitrogen (Carlsbad, CA, USA).

### 2.3. Virus Preparation, Titration and Infection

HCMV (AD169) and MCMV (Smith strain) stocks were prepared in MRC-5 cells and MEF cells, and aliquots were stored at −80 °C. Viral titers were determined using the 50% tissue culture infective dose (TCID_50_) method, as previously described [26]. Briefly, HFF or MEF cells were incubated with mock, UV-inactivated HCMV (UV-HCMV) or HCMV for 1 h under serum free DMEM at a multiplicity of infection (MOI) of 0.5. Then, the medium was removed, the cells were washed with PBS and fresh serum free medium was added. All experiments were examined at least three times using Reed and Muench’s method.

### 2.4. DCF Staining

The measurement of reactive oxygen species (ROS) production in response to H_2_O_2_ was performed, as previously described [27]. After treatment with H_2_O_2_ (0, 100, or 200 μM) for 24 h, cells were washed with PBS and incubated with 10 μM H_2_DCF-DA in warmed, serum free DMEM for 30 min in a CO_2_ incubator at 37 °C. Cells were then washed three times with PBS and images were taken using a Leica microscope.

### 2.5. Detection of Cellular Catalase Activity and Intracellular H_2_O_2_ Level

HFF cells cultured with H_2_O_2_ (0, 100 or 200 μM) or ATA (0, 1.0, 2.0 or 4.0 mM) for 24 h were harvested by centrifugation. The cell pellets were sonicated in 100 μL cold PBS. After centrifugation at 10,000× *g* and 4 °C for 5 min, the supernatants were used for the detection of catalase activity and intracellular H_2_O_2_ level. The assay kits were all purchased from Jiancheng Bioengineering Institute (Nanjing, China).

### 2.6. Luciferase Assays

HEK 293 cells were transiently transfected with the MIEP-pGL3 luciferase reporter plasmid and the pRL-TK vector. At 12 h after transfection, the cells were treated with or without the antioxidants NAC (5 mM), l-glutathione (5 mM), and catalase (800 U/mL) for 2 h and then stimulated for 24 h with H_2_O_2_ (200 μM) or ATA (4 mM), or were treated with H_2_O_2_ (0, 50, 100, or 200 μM) or ATA (0, 1, 2, or 4 mM) alone for 24 h. Luciferase activity was determined as previously described [25] using the Dual-Luciferase^®^ Reporter Assay System (Promega, Madison, WI, USA).

### 2.7. Real-Time PCR

Total DNA was isolated from the supernatants of infected cells using a cell and tissue genomic DNA extraction kit (BioTeke Corporation, Beijing, China). Changes in viral DNA loads were monitored using absolute quantitative real-time PCR. Viral DNA levels were detected using primers against the HCMV IE1 gene (forward primer, 5′-ATGTACGGGGGCATCTCTCT-3′ and reverse primer, 5′-GGCTTGGTTATCAGAGGCCG-3′) or the MCMV IE1 gene (forward primer, 5′-GTGGGCATGAAGTGTGGGTA-3′ and reverse primer, 5′-CGCATCGAAAGACAACGCAA-3′).

### 2.8. qRT-PCR

Total RNA was extracted using TRIzol reagent (Invitrogen) at 24 h after HCMV infection (MOI = 0.5). cDNA was prepared using ReverTra Ace^®^ qRCR RT Master Mix with gDNA Remover (TOYOBO, Osaka, Japan). Each sample was measured in triplicate. The expression level of the IE1 gene transcript (forward primer, 5′-GTTGGCCGAAGAATCCCTCA-3′ and reverse primer, 5′-CACCATGTCCACTCGAACCT-3′) was normalized to GAPDH mRNA (forward primer, 5′-CATGAGAAGTATGACAACAGCCT-3′ and reverse primer, 5′-AGTCCTTCCACGATACCAAAGT-3′). Compared to the untreated cells, the relative expression levels in treated cells were calculated as fold changes.

### 2.9. Western Blot Analysis

Cells pellets were lysed in lysis buffer (Promega) with a cocktail of protein inhibitors (Roche, Mannheim, Germany) and then centrifuged at 13,000× *g* and 4 °C for 10 min. In brief, 30 μg whole cell extract was heated for 5 min at 98 °C with Laemmli buffer, and then, samples were separated by 12% sodium dodecyl sulfate polymerase gel electrophoresis (SDS-PAGE) and transferred to polyvinylidene difluoride (PVDF) membranes (Millipore, Billerica, MA, USA). After blocking in 5% (*w/v*) skim milk (Applygen, Beijing, China) or 5% (*w/v*) bovine serum albumin (BSA) (MP, Auckland, New Zealand), the blots were probed with primary antibodies overnight at 4 °C. Protein bands were detected using Western blotting luminol reagent (Santa Cruz Biotechnology). The membranes were incubated with Western Blot stripping buffer (CWBio) to re-probe for other proteins in the same membrane.

### 2.10. Animal Studies

BALB/c mice (male, 6–8 weeks old, 20–25 g body weight) were purchased from Vital River (Beijing, China). The animal study was performed according to the protocols approved by the Ethics Committee at the Beijing Institute of Transfusion Medicine and in accordance with Institutional Animal Care and Use Committee (IACUC) guidelines.

Mice were treated intragastrically with 400 μL of 40 mM NAC in water every day, from 3 days before intraperitoneal inoculation with MCMV (Smith strain, 5 × 10^3^ p.f.u). At the proper time (day 7, 14, 21 and 28 post infection), DNA was extracted from 100 μL whole blood and used to determine the viral DNA load. To detect infectious virions in mice organs, the salivary glands (50 mg) and the lung (50 mg) were collected and homogenized on day 14 and 28, and viral titer was calculated with TCID_50_ assays in MEF monolayers.

### 2.11. Statistical Analysis

All values are expressed as the means ± standard deviations. Statistical analyses were performed using SPSS statistical software V.17 (SPSS Inc., Chicago, IL, USA). Significant differences were evaluated by the two-tailed Student’s *t*-test when two groups were compared, one-way analysis of variance (ANOVA) followed by the Dunnett’s test when multiple groups were tested against a control group and the Bonferroni *post hoc* test when performing multiple comparisons between groups. A *p*-value lower than 0.05 was considered to indicate a statistically significant difference.

## 3. Results

### 3.1. ROS Enhance HCMV Replication through Paracrine and Autocrine Mechanisms

**Figure 1:**
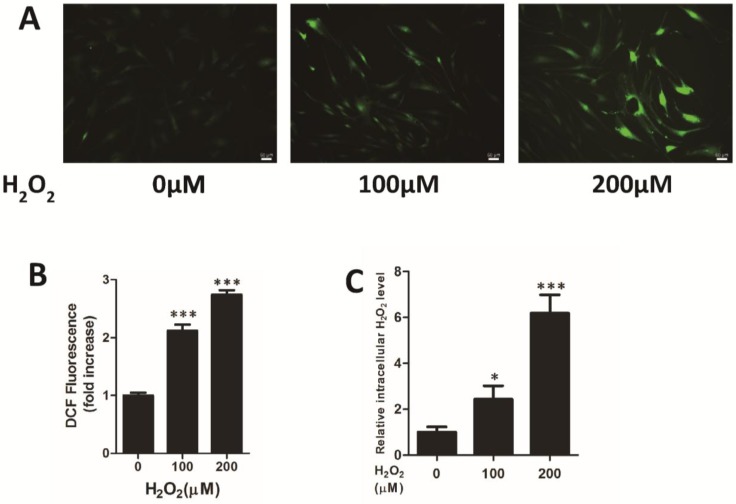
Oxidative stress was induced in human fibroblast cells by treatment with H_2_O_2_. After supplementation with 0, 100, 200 μM H_2_O_2_ for 24 h, increasing reactive oxygen species (ROS) production in human foreskin fibroblast (HFF) cells was determined by staining (**A**) or by measuring (**B**) the fluorescence produced after a 30 min incubation at 37 °C with 10 μM 2′,7′-dichlorodihydrofluorescein diacetate (H_2_DCFH-DA) (for total ROS). (**C**) Treatment of HFF cells with 0, 100, 200 μM H_2_O_2_ increased the intracellular H_2_O_2_ concentration. The data are expressed as the means ± SD. * *p* < 0.05 or *** *p* < 0.001 for treated cells *versus* untreated cells by Dunnett’s test.

After treatment with exogenous hydrogen peroxide for 24 h, HFF cells dose-dependently formed an increasing amount of ROS (Figure 1A,B) and H_2_O_2_ content (Figure 1C).

**Figure 2:**
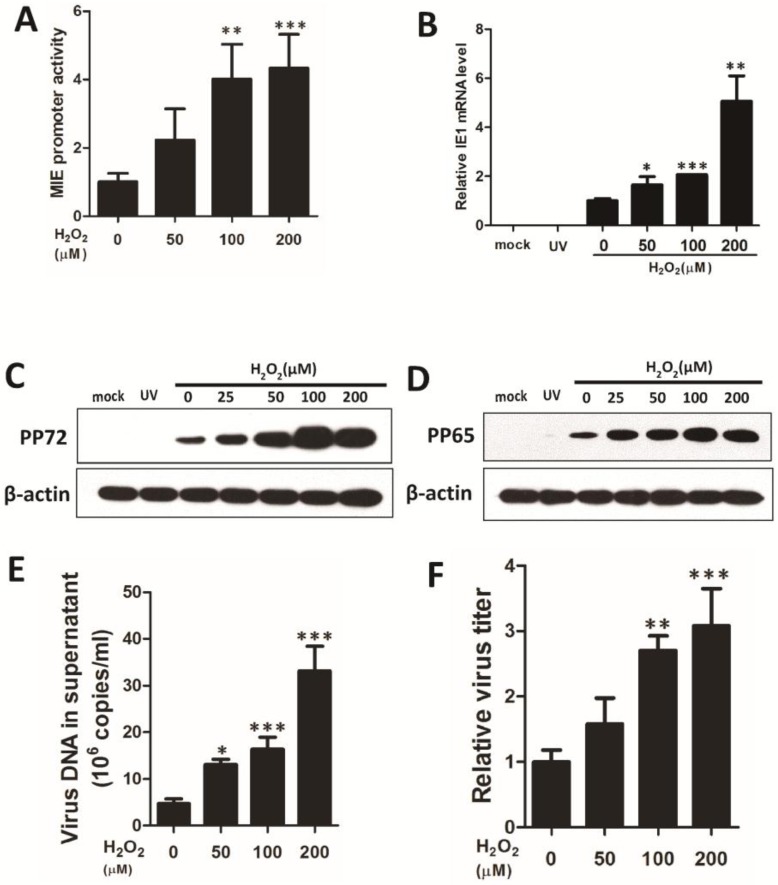
Exogenous H_2_O_2_ induces HCMV replication in HFF cells. Luciferase activities were measured for 293 cells transfected with promoter reporter plasmids for 24 h and treated with H_2_O_2_ (0, 50, 100, 200 μM) for 12 h (**A**). Cells were infected with UV-HCMV (UV) or HCMV at a multiplicity of infection (MOI) of 0.5. Incubation with exogenous H_2_O_2_ (0, 50, 100, or 200 μM) for 24 h induced the expression of immediately early (IE1) transcript in HCMV infected cells (**B**). Proteins were collected from infected cells for treatment with 0, 25, 50, 100, 200 μM H_2_O_2_ for 72 h. Viral proteins pp72 (**C**) and pp65 (**D**) were detected by Western blotting using β-actin for calibration of sample loading. Viral DNA load and viral titer and were measured after a 72 h treatment with 0, 50, 100, or 200 μM H_2_O_2_ by real-time quantitative PCR (**E**) and TCID_50_ assay (**F**). * *p* < 0.05; ** *p* < 0.01 or *** *p* < 0.001 for treated *versus* untreated cells.

To investigate the role of H_2_O_2_ in HCMV lytic replication, we observed whether exogenous H_2_O_2_ is sufficient to enhance HCMV replication at both the mRNA and protein level. HCMV MIE promoter activities were induced by H_2_O_2_ (Figure 2A), and H_2_O_2_ increased the expression of the IE gene in a dose-dependent manner (Figure 2B). Furthermore, H_2_O_2_ increased the levels of viral proteins, including pp72 and pp65 (Figure 2C,D), as well as the production of HCMV DNA in the culture supernatant (Figure 2E) and infectious virions (Figure 2F).

**Figure 3:**
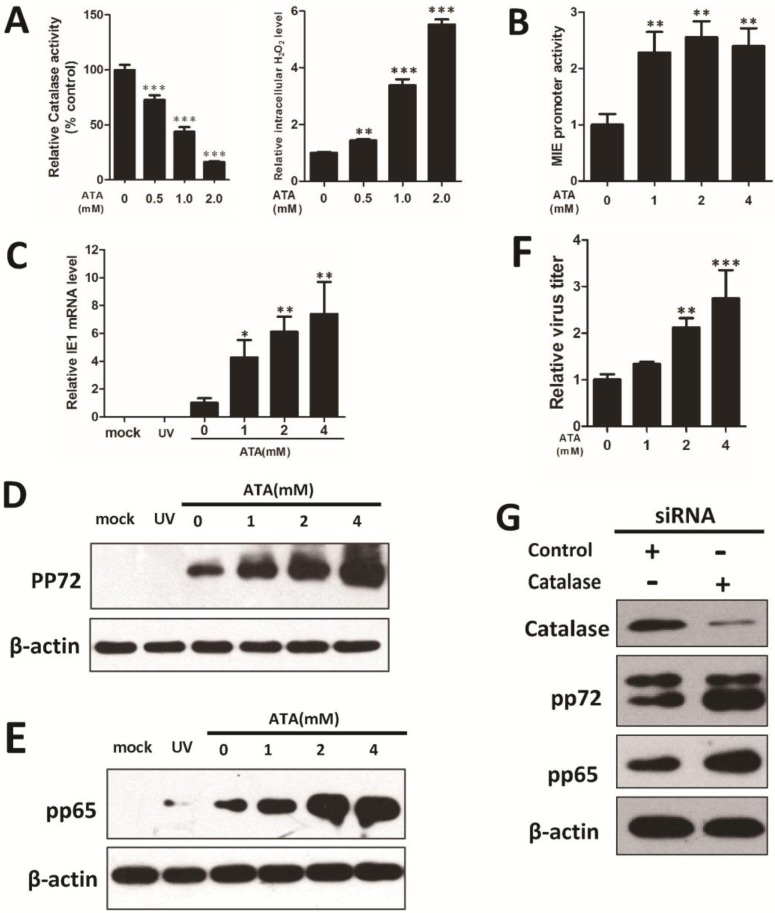
ATA-induced intracellular H_2_O_2_, enhancing viral replication in HFF cells. Treatment of HFF cells with the catalase inhibitor 3-amino-1,2,4-triazole (ATA) (0, 1, 2, 4 mM) for 24 h reduced catalase activity and increased intracellular H_2_O_2_ level in a dose-dependent manner (**A**). Cells were cultured with ATA for 24 h. ATA-induced intracellular H_2_O_2_ increased MIE promoter activity (**B**) and HCMV IE1 transcription (**C**). Cells were infected with UV-HCMV (UV) or HCMV at an MOI of 0.5. An increase in pp72 (**D**) and pp65 (**E**) protein levels were detected by Western blotting under 0, 1, 2, 4 mM ATA treatments for 72 h, and β-actin was used to calibrate sample loading. (**F**) Relative virus titers were measured using TCID_50_ assay within five days. (**G**) Catalase, HCMV lytic protein pp72, pp65 and β-actin levels were determined by Western blotting after treatment with siRNA for five days. * *p* < 0.05; ** *p* < 0.01 or *** *p* < 0.001 for ATA-treated *versus* untreated cells.

Next, we verified whether an increase in intracellular H_2_O_2_ is sufficient to induce HCMV replication. Treatment of HFF cells with ATA, an inhibitor of the H_2_O_2_-scavenging enzyme catalase, reduced the activity of cellular catalase (Figure 3A left panel) and increased the intracellular H_2_O_2_ level (Figure 3A right panel). ATA increased the MIE promoter activities, the IE1 gene transcripts, the expression of HCMV pp72 and pp65, and production of infectious virions (Figure 3B–F).

To confirm that the effect of ATA on HCMV replication was the result of an increase in the intracellular level of H_2_O_2_, we transiently expressed a catalase-specific siRNA in HFF cells. Compared to cells expressing a control siRNA, those transfected with a catalase-specific specific siRNA showed greatly lower catalase protein expression. Similar to ATA treatment, knockdown of catalase enhanced HCMV lytic pp72 and pp65 protein levels (Figure 3G).

### 3.2. H_2_O_2_ Scavengers Inhibit H_2_O_2_-Upregulated HCMV Lytic Replication

**Figure 4:**
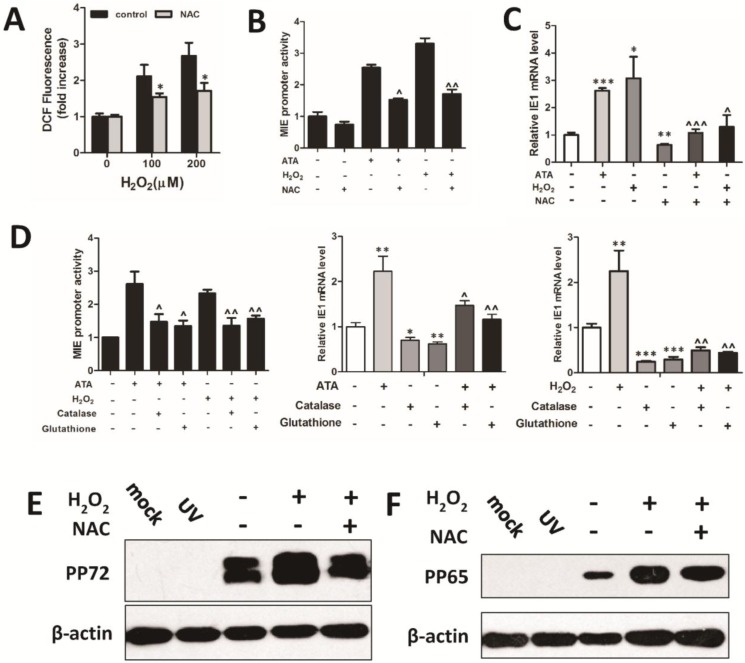

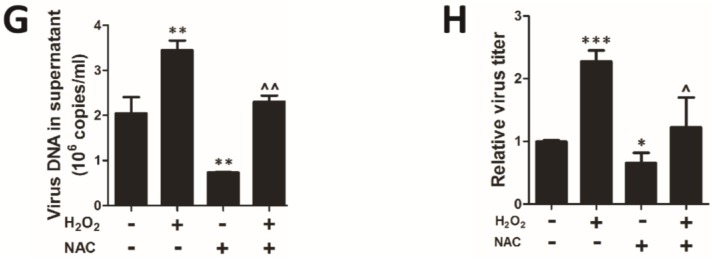
H_2_O_2_ scavengers inhibit H_2_O_2_-induced HCMV lytic replication *in vitro*. Treatment with the H_2_O_2_ scavenger *N*-acetylcysteine (NAC) (5 mM) was shown to decrease ROS production in HFF cells by measurement of fluorescence (**A**). Cells were treated with 200 μM H_2_O_2_ without any scavengers, or with the scavenger NAC at 5 mM, catalase at 800 U/mL or reduced glutathione at 5 mM for 24 h and then HCMV infection (MOI = 0.5). H_2_O_2_ scavengers reduced H_2_O_2_ (200 μM) and ATA (4 mM)-induction of MIE promoter activity and IE transcription in HFF cells (**B**,**C**). Treated with 200 μM H_2_O_2_ upregulated HCMV lytic replication, but inhibited by treatment with 5 mM NAC. pp72 and pp65 viral proteins in UV-HCMV (UV) or HCMV infected HFF cells treated with NAC (5 mM) for 72 h were determined by Western blotting with β-actin to calibrate sample loading (**E**,**F**). Treatment with 5 mM NAC downregulated viral DNA load (**G**) in culture supernatants and viral titer (**H**) in the presence or absence of 200 μM H_2_O_2_. * *p* < 0.05; ** *p* < 0.01 or *** *p* < 0.001 for treated *versus* untreated cells. ^ *p* < 0.05; ^^ *p* < 0.01 or ^^^ *p* < 0.001 for H_2_O_2_ scavenger-treated cells *versus* H_2_O_2_- or ATA-treated cells.

To detect whether H_2_O_2_ is required for HCMV lytic replication, we used a H_2_O_2_ scavenger to decrease the intracellular H_2_O_2_ level. Treatment with NAC, a common H_2_O_2_ scavenger, decreased the cellular H_2_O_2_ level, as indicated by a reduction in the median fluorescent level, in H_2_-DCFH-treated HFF cells (Figure 4A). As expected, NAC inhibited the upregulation of MIE promoter activities (Figure 4B) and IE1 transcription (Figure 4C) by supplementation with ATA and H_2_O_2_. The effects of H_2_O_2_ on upregulation of HCMV MIE promoter activity and viral gene expression were also inhibited by another two scavengers, catalase and reduced glutathione (Figure 4D). Furthermore, NAC impaired the H_2_O_2_-upregulated HCMV lytic protein and infectious virions (Figure 4E–H). These results indicate that H_2_O_2_ scavengers, such as NAC and catalase, can suppress the stimulation of HCMV lytic replication by H_2_O_2_.

### 3.3. H_2_O_2_ Scavenger NAC Inhibits MCMV Lytic Replication *in Vivo*

**Figure 5:**
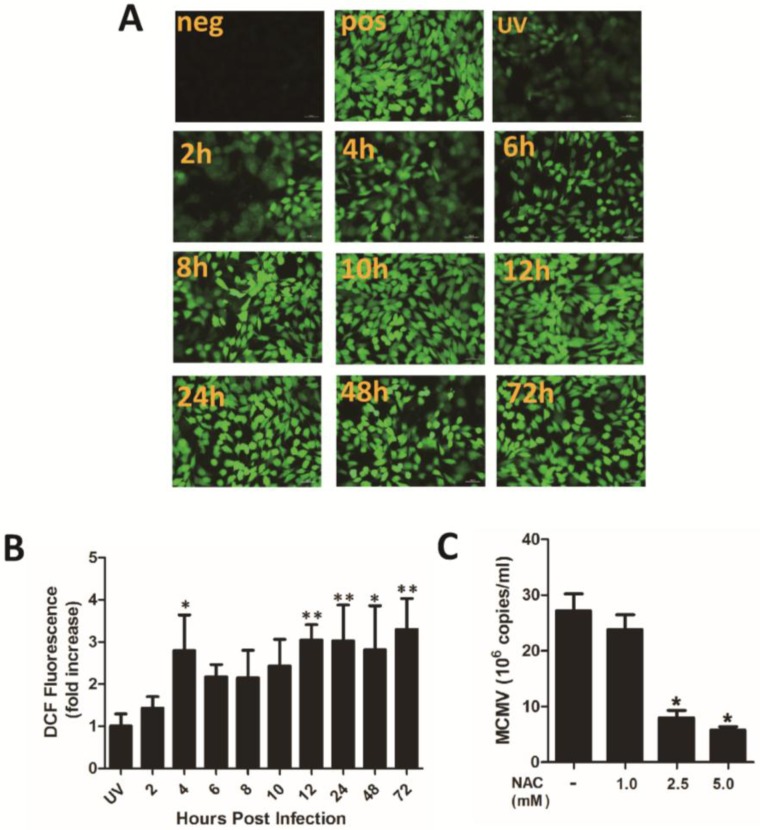

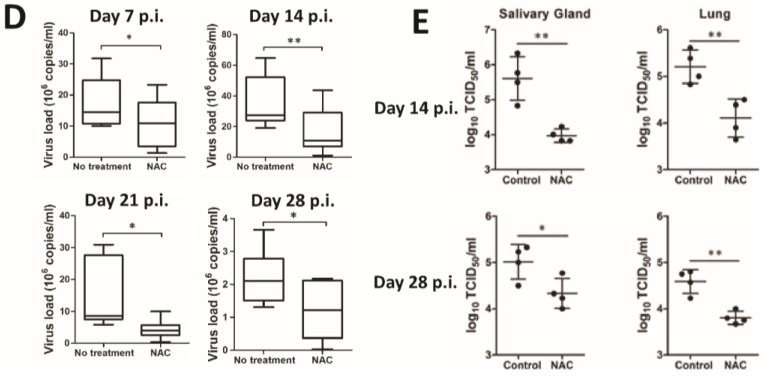
The H_2_O_2_ scavenger NAC inhibits MCMV lytic replication *in vivo*. ROS production upon primary infection of MEF cells with MCMV. Confluent MEF cells in 24-well plates were serum starved for 2 h, incubated with DMEM containing 10 μM H_2_-DCFDA for 30 min at 37 °C, and infected with either UV-inactivated MCMV or MCMV (MOI = 0.5). H_2_-DCFDA fluorescence were stained between infected and uninfected cells at indicated times (**A**). Fold induction of ROS production in infected cells relative to UV-HCMV (UV) infected cells (**B**). The culture supernatant collected at 72 h post infection with MCMV at an MOI of 0.5 detected the viral DNA (**C**). Mice were treated intragastrically with 400 μL of 40 mM NAC in water every day, from three days before intraperitoneal inoculation with MCMV (Smith strain, 5 × 10^3^ p.f.u). One hundred microliters of whole blood from each mouse were examined. Viral loads in blood samples of control (*n* = 14) and NAC-treated (*n* = 14) mice at indicated days post infection (**D**). Related infectious viral titer in the salivary glands and in the lung was detected at 14 and 28 days post infection by TCID_50_ assay (**E**). * *p* < 0.05 or ** *p* < 0.01 for treated *versus* untreated cells and mice.

Because of the strict species specificity of CMV, it is difficult to establish an animal model of HCMV infection. MCMV, which is similar to HCMV biological characteristics [28,29], has been regularly used to mimic HCMV infection *in vitro* and *in vivo*. We sought to inhibit MCMV lytic replication in mice using H_2_O_2_ scavenger NAC. We found that oxidative stress production was induced during primary infection of MEF cells with MCMV (Figure 5A,B). Conversely, supplement with NAC strongly inhibited MCMV infection of MEF (Figure 5C).

To examine CMV lytic replication and verify the inhibitory effect of antioxidant NAC *in vivo*, we treated BALB/c mice intragastrically with 400 μL of 40 mM NAC. The viral DNA load in whole blood was measured on day 7, 14, 21, and 28 post-infection. We found that mice fed NAC had a lower viral load than those fed drinking water alone (Figure 5D). To determine the production of infectious virions in mice organs, we used cell-free supernatants from ultrasonic homogenates of the salivary glands and the lung to infect MEF. We observed a high viral titer in cells infected with supernatants from the control group, while those infected with supernatants from NAC-treated mice had lower ones on days 14 and 28, respectively (Figure 5E). Collectively, the results of these *in vivo* experiments indicate that the antioxidant NAC effectively decreased MCMV replication in these mice.

### 3.4. H_2_O_2_ Upregulates HCMV Replication by Activating the p38 MAPK Pathway

**Figure 6:**
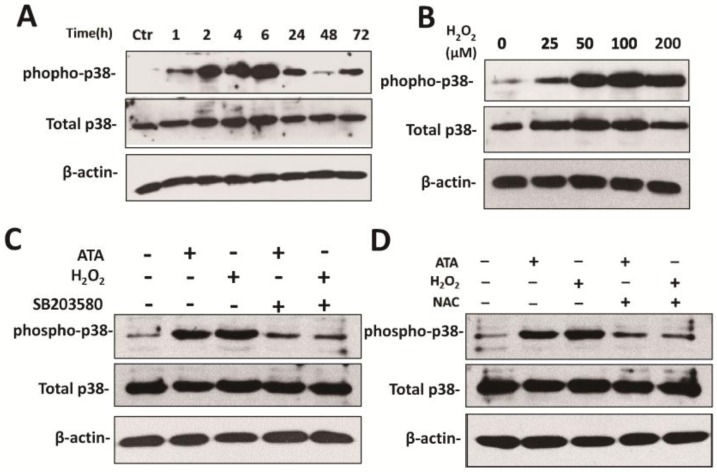

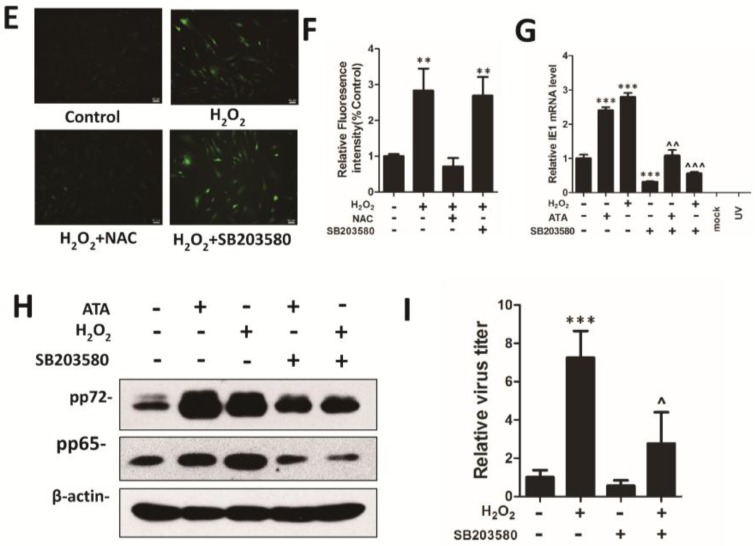
H_2_O_2_ facilitates HCMV replication by activating the p38-MAPK pathway. HFF cells were left untreated or were treated with 200 μM H_2_O_2_ for the indicated times (**A**) or with various H_2_O_2_ concentrations for 6 h (**B**). The kinases were detected by Western blotting, using specific primary antibodies against phospho-p38 and p38 and β-actin to calibrate sample loading. HFF cells were treated with SB203580 (10 μM) or NAC (5 mM) 1 h prior to H_2_O_2_ (200 μM) and ATA (5 mM) stimulation. Cells were harvested at 6 h post H_2_O_2_ and ATA treatment (**C**,**D**). Cells were determined by staining (**E**) or by measuring (**F**) the fluorescence produced after a 30 min incubation at 37 °C with 10 μM H_2_DCFH-DA. Cells were infected with UV-HCMV (UV) or HCMV at an MOI of 0.5. Real-time PCR analysis of IE1 mRNA levels in cells allowed comparisons to untreated cells. Total mRNA was extracted from HFF at 24 h post infection. At 72 h, pp72 and pp65 protein were detected by Western blotting (**H**). Viral titer was detected in the presence or absence of H_2_O_2_ under a treatment with 10 μM SB203580 (**I**). ** *p* < 0.01 or *** *p* <0.001 for treated *versus* untreated cells. ^ *p* < 0.05; ^^ *p* < 0.01 or ^^^ *p* < 0.001 for p38 inhibitor SB203580-treated cells *versus* H_2_O_2_ or ATA-treated cells.

As previously presented (Figure 2), H_2_O_2_ stimulates the upregulation of HCMV replication, but the mechanism is unclear. Here, the results revealed that p38-MAPK was rapidly and strongly activated by H_2_O_2_ treatment, following a time- and dose-dependent pattern (Figure 6A,B). In particular, p38-MAPK activation displayed a rapid onset within 1 h of treatment, followed by a progressive increase, returning to basal levels within 48 h, while a second peak was observed at 72 h after treatment (Figure 6A). Increasing H_2_O_2_ concentration led to an increase of p38-MAPK phosphorylation (Figure 6B) and the minimal concentration of H_2_O_2_ was 25 μM. Next, treatment with 10 μM SB203580 reduced both H_2_O_2_- and ATA-activated p38 phosphorylation (Figure 6C). Since supplementation with NAC inhibited H_2_O_2_- and ATA-induced HCMV replication (Figure 5), we suppose that NAC could inhibit H_2_O_2_-induced p38-MAPK activation. As expected, pretreatment with NAC (5 mM) also strongly decreased ATA- and H_2_O_2_-induced activation of p38-MAPK (Figure 6D). This effect was consistent with a decline of H_2_O_2_-induced oxidative stress in cells (Figure 6E,F), while SB203580 inhibited the H_2_O_2_-induced activation of p38 without affecting the redox status. At the same time, the upregulation of IE1 gene transcription, the expression of viral pp72 and pp65 proteins and the production of infectious virions were inhibited by treatment with SB203580 (Figure 6G–I). These results indicated that H_2_O_2_ upregulation of HCMV replication was mediated by the p38 MAPK pathway and that the inhibitory effect of NAC on H_2_O_2_-induced HCMV replication was also involved in this pathway.

## 4. Discussion

In this study, we investigated the role of H_2_O_2_ in the regulation of viral lytic replication in HFF. We demonstrate that hydrogen peroxide upregulates HCMV lytic replication through extracellular and intracellular mechanisms in fibroblasts. In addition, pretreatment with antioxidants inhibits HCMV replication *in vitro* and *in vivo*. Mechanistically, the p38-MAPK pathway contributes to the stimulation of HCMV replication by H_2_O_2_.

Since AIDS, organ transplantation, and atherosclerosis are characterized by oxidative stress, and HCMV infection is of high morbidity and mortality among these patients, oxidative stress might be a crucial physiological factor that upregulates HCMV lytic replication in these cases. Regularly, the reactive oxygen species H_2_O_2_ is often used to induce intracellular oxidative stress *in vitro* [30] and our results demonstrated that treatment with exogenous H_2_O_2_ for 24 h impairs cellular redox homeostasis and acted as an active ROS in cells (Figure 1). ATA, a small molecule irreversible inhibitor of the H_2_O_2_-scavenging enzyme catalase, was utilized to induce intracellular H_2_O_2_ in cells (Figure 3A) and thereby performed the same role as exogenous H_2_O_2_ (Figure 3B–F).

HCMV lytic replication is initiated by IE1 transcription, which is activated by MIE promoter/enhancer activity. Studies have shown that ROS can enhance IE transcription products in human endothelial cells and smooth muscle cells [31,32], but this has rarely been declared in fibroblasts. Interestingly, it has been shown that oxidative stress can lead to the reactivation and replication of KSHV, another member of the herpesvirus family, in PEL cells and endothelial cells [33,34]. The luciferase reporter assay showed that treatment with H_2_O_2_ enhances the activity of HCMV MIE promoter in a dose-dependent manner. Consistent with this result, we detected an increasing expression of viral IE1 gene and production of virions under the treatment of both H_2_O_2_- and ATA-induced oxidative stress. These results indicated that viral gene transcription and viral replication in the permissible cells, HFF, was initiated by H_2_O_2_-upregulated HCMV major immediately promoter activity.

Cellular antioxidants, such as superoxide dismutase (SOD) and catalase (CAT), protect cells from oxidative stress. SOD catalyzes the transition of superoxide into H_2_O_2_, which can be further converted into H_2_O and O_2_ by catalase. Antioxidants used to inhibit oxidative stress have been shown to block the replication of RNA viruses, including influenza virus, EV71, and HIV-1 [35,36]. However, it remains unclear whether H_2_O_2_ scavengers can inhibit H_2_O_2_-induced HCMV replication. In this study, NAC treatment resulted in inhibition of H_2_O_2_-induced oxidative stress and H_2_O_2_-upregulated HCMV replication. Similar to NAC, catalase and reduced glutathione also inhibited H_2_O_2_ induced MIE promoter transcription and IE gene expression. In addition, we illustrated that ROS were required for upregulation of HCMV replication induced by CAT inhibition and depletion. These results support the hypothesis that the reactive oxygen species hydrogen peroxide is a key factor in the enhancement of HCMV gene expression and replication and that this effect could be inhibited by treatment with H_2_O_2_ scavengers *in vitro*. Therefore, our results exhibited a critical role for H_2_O_2_ and cellular antioxidants in regulating HCMV replication.

Accumulating evidence has suggested an essential role for oxidative stress during viral infection [37,38,39,40,41]. Oxidative stress can be considered a protective means of the cell, which can contribute to apoptosis [42], and thus prevent the virus from replicating and infecting other cells. Interestingly, CMV appears to utilize virus-specific mechanisms to protect the cell from the effects of ROS and maintain a redox homeostasis [43]. However, the results showed that the levels of ROS increased remarkably upon MCMV infection, with the increase first appearing at approximately 2 h after infection (Figure 5A) and sustained until 72 h post-infection. (Figure 5B). Thus, it seems antioxidant therapy could be a potential treatment method for primary MCMV infection. In support of the results of previous studies we conducted, NAC was shown to prevent MCMV replication and production *in vitro* (Figure 5C). Significantly, NAC strongly reduced MCMV DNA load in whole blood and the production of infectious virions in the salivary gland and the lung.

It is widely accepted that the HCMV major immediate early promoter contains several types of transcription factor binding sites [44], such as NF-κB, that can be induced by H_2_O_2_ [45]. Furthermore, previous studies have shown that H_2_O_2_ can induce NF-κB transcription through multiple signaling pathways [23,46], including the JNK and p38 MAPK pathways. Furthermore, studies have shown that p38 MAPK, which is mediated by MSK1, is involved in NF-κB transactivation by H_2_O_2_ stimulus [23,24], but very little is known regarding the possible linkage between this pathway under H_2_O_2_-upregulated HCMV replication in fibroblast cells. Here, consistent with the results of previous studies, we showed that p38 was rapidly and strongly activated by H_2_O_2_ treatment. Additionally, co-culturing fibroblasts with HCMV and the p38 specific inhibitor SB203580 decreased the phosphorylation of p38 and HCMV transcription and production. Similar to SB203580, NAC also hampered the activation of p38 by H_2_O_2_ and inhibited the viral replication. However, NAC inhibited the H_2_O_2_-induced phosphorylation of p38 through inhibition of H_2_O_2_-induced oxidative stress, while SB203580 directly inhibited the p38 activation without affecting the production of ROS. Thus, we first demonstrated that H_2_O_2_ induced HCMV replication through the ROS/P38 MAPK signaling pathway.

Conclusively, our findings suggest that further studies of the antiviral and immune-modulatory effects of antioxidants are warranted. Furthermore, targeting of hydrogen peroxide and H_2_O_2_-mediated signaling is a potential therapeutic or preventive approach in HCMV infection.

## References

[1] Gerna G., Baldanti F., Revello M.G. (2004). Pathogenesis of human cytomegalovirus infection and cellular targets. Hum. Immunol..

[2] Rubin R.H. (1990). Impact of cytomegalovirus infection on organ transplant recipients. Rev. Infect. Dis..

[3] Patel R., Snydman D.R., Rubin R.H., Ho M., Pescovitz M., Martin M., Paya C.V. (1996). Cytomegalovirus prophylaxis in solid organ transplant recipients. Transplantation.

[4] Castro-Malaspina H., Harris R.E., Gajewski J., Ramsay N., Collins R., Dharan B., King R., Deeg H.J. (2002). Unrelated donor marrow transplantation for myelodysplastic syndromes: Outcome analysis in 510 transplants facilitated by the National Marrow Donor Program. Blood.

[5] Steininger C., Puchhammer-Stockl E., Popow-Kraupp T. (2006). Cytomegalovirus disease in the era of highly active antiretroviral therapy (HAART). J. Clin. Virol..

[6] Weis M., Kledal T.N., Lin K.Y., Panchal S.N., Gao S.Z., Valantine H.A., Mocarski E.S., Cooke J.P. (2004). Cytomegalovirus infection impairs the nitric oxide synthase pathway: Role of asymmetric dimethylarginine in transplant arteriosclerosis. Circulation.

[7] Simmonds J., Fenton M., Dewar C., Ellins E., Storry C., Cubitt D., Deanfield J., Klein N., Halcox J., Burch M. (2008). Endothelial dysfunction and cytomegalovirus replication in pediatric heart transplantation. Circulation.

[8] Arasaratnam R.J. (2013). Cytomegalovirus and cardiovascular disease--the importance of covariates. J. Infect. Dis..

[9] Rahbar A., Bostrom L., Lagerstedt U., Magnusson I., Soderberg-Naucler C., Sundqvist V.A. (2003). Evidence of active cytomegalovirus infection and increased production of IL-6 in tissue specimens obtained from patients with inflammatory bowel diseases. Inflamm. Bowel Dis..

[10] Reeves M.B., MacAry P.A., Lehner P.J., Sissons J.G., Sinclair J.H. (2005). Latency, chromatin remodeling, and reactivation of human cytomegalovirus in the dendritic cells of healthy carriers. Proc. Natl. Acad. Sci. USA.

[11] Reeves M.B., Compton T. (2011). Inhibition of inflammatory interleukin-6 activity via extracellular signal-regulated kinase-mitogen-activated protein kinase signaling antagonizes human cytomegalovirus reactivation from dendritic cells. J. Virol..

[12] Reeves M.B., Breidenstein A., Compton T. (2012). Human cytomegalovirus activation of ERK and myeloid cell leukemia-1 protein correlates with survival of latently infected cells. Proc. Natl. Acad. Sci. USA.

[13] Rodems S.M., Spector D.H. (1998). Extracellular signal-regulated kinase activity is sustained early during human cytomegalovirus infection. J. Virol..

[14] Johnson R.A., Ma X.L., Yurochko A.D., Huang E.S. (2001). The role of MKK1/2 kinase activity in human cytomegalovirus infection. J. Gen. Virol..

[15] Jassem W., Fuggle S.V., Rela M., Koo D.D., Heaton N.D. (2002). The role of mitochondria in ischemia/reperfusion injury. Transplantation.

[16] Kedzierska K., Sporniak-Tutak K., Bober J., Safranow K., Olszewska M., Jakubowska K., Domanski L., Golembiewska E., Kwiatkowska E., Laszczynska M. (2011). Oxidative stress indices in rats under immunosuppression. Transplant. Proc..

[17] Lamoureux F., Mestre E., Essig M., Sauvage F.L., Marquet P., Gastinel L.N. (2011). Quantitative proteomic analysis of cyclosporine-induced toxicity in a human kidney cell line and comparison with tacrolimus. J. Proteomics.

[18] Sharma B. (2014). Oxidative stress in HIV patients receiving antiretroviral therapy. Curr. HIV Res..

[19] Pastori D., Carnevale R., Pignatelli P. (2014). Is there a clinical role for oxidative stress biomarkers in atherosclerotic diseases?. Intern. Emerg. Med..

[20] Li H., Horke S., Forstermann U. (2014). Vascular oxidative stress, nitric oxide and atherosclerosis. Atherosclerosis.

[21] Pan J.S., Hong M.Z., Ren J.L. (2009). Reactive oxygen species: A double-edged sword in oncogenesis. World J. Gastroenterol..

[22] Sinclair J. (2010). Chromatin structure regulates human cytomegalovirus gene expression during latency, reactivation and lytic infection. Biochim. Biophys. Acta.

[23] Aggeli I.K., Gaitanaki C., Beis I. (2006). Involvement of JNKs and p38-MAPK/MSK1 pathways in H2O2-induced upregulation of heme oxygenase-1 mRNA in H9c2 cells. Cell. Signal..

[24] Kefaloyianni E., Gaitanaki C., Beis I. (2006). ERK1/2 and p38-MAPK signalling pathways, through MSK1, are involved in NF-kappaB transactivation during oxidative stress in skeletal myoblasts. Cell. Signal..

[25] Wen D.Q., Zhang Y.Y., Lv L.P., Zhou X.P., Yan F., Ma P., Xu J.B. (2009). Human cytomegalovirus-encoded chemokine receptor homolog US28 stimulates the major immediate early gene promoter/enhancer via the induction of CREB. J. Recept. Signal Transduct. Res..

[26] Keyes L.R., Bego M.G., Soland M., St Jeor S. (2012). Cyclophilin A is required for efficient human cytomegalovirus DNA replication and reactivation. J. Gen. Virol..

[27] Satoh K., Nigro P., Matoba T., O’Dell M.R., Cui Z., Shi X., Mohan A., Yan C., Abe J., Illig K.A. (2009). Cyclophilin A enhances vascular oxidative stress and the development of angiotensin II-induced aortic aneurysms. Nat. Med..

[28] Rawlinson W.D., Farrell H.E., Barrell B.G. (1996). Analysis of the complete DNA sequence of murine cytomegalovirus. J. Virol..

[29] Krmpotic A., Bubic I., Polic B., Lucin P., Jonjic S. (2003). Pathogenesis of murine cytomegalovirus infection. Microbes Infect./Inst. Pasteur.

[30] Bak M.J., Jeong W.S., Kim K.B. (2014). Detoxifying effect of fermented black ginseng on H2O2-induced oxidative stress in HepG2 cells. Int. J. Mol. Med..

[31] Scholz M., Cinatl J., Gross V., Vogel J.U., Blaheta R.A., Freisleben H.J., Markus B.H., Doerr H.W. (1996). Impact of oxidative stress on human cytomegalovirus replication and on cytokine-mediated stimulation of endothelial cells. Transplantation.

[32] Speir E., Shibutani T., Yu Z.X., Ferrans V., Epstein S.E. (1996). Role of reactive oxygen intermediates in cytomegalovirus gene expression and in the response of human smooth muscle cells to viral infection. Circ. Res..

[33] Li X., Feng J., Sun R. (2011). Oxidative stress induces reactivation of Kaposi’s sarcoma-associated herpesvirus and death of primary effusion lymphoma cells. J. Virol..

[34] Ye F., Zhou F., Bedolla R.G., Jones T., Lei X., Kang T., Guadalupe M., Gao S.J. (2011). Reactive oxygen species hydrogen peroxide mediates Kaposi's sarcoma-associated herpesvirus reactivation from latency. PLoS Pathog..

[35] Cai J., Chen Y., Seth S., Furukawa S., Compans R.W., Jones D.P. (2003). Inhibition of influenza infection by glutathione. Free Radic. Biol. Med..

[36] Staal F.J., Roederer M., Herzenberg L.A., Herzenberg L.A. (1990). Intracellular thiols regulate activation of nuclear factor kappa B and transcription of human immunodeficiency virus. Proc. Natl. Acad. Sci. USA.

[37] McGuire K.A., Barlan A.U., Griffin T.M., Wiethoff C.M. (2011). Adenovirus type 5 rupture of lysosomes leads to cathepsin B-dependent mitochondrial stress and production of reactive oxygen species. J. Virol..

[38] Barlan A.U., Griffin T.M., McGuire K.A., Wiethoff C.M. (2011). Adenovirus membrane penetration activates the NLRP3 inflammasome. J. Virol..

[39] Tung W.H., Hsieh H.L., Lee I.T., Yang C.M. (2011). Enterovirus 71 induces integrin beta1/EGFR-Rac1-dependent oxidative stress in SK-N-SH cells: Role of HO-1/CO in viral replication. J. Cell. Physiol..

[40] Kavouras J.H., Prandovszky E., Valyi-Nagy K., Kovacs S.K., Tiwari V., Kovacs M., Shukla D., Valyi-Nagy T. (2007). Herpes simplex virus type 1 infection induces oxidative stress and the release of bioactive lipid peroxidation by-products in mouse P19N neural cell cultures. J. Neurovirol..

[41] Aubert M., Chen Z., Lang R., Dang C.H., Fowler C., Sloan D.D., Jerome K.R. (2008). The antiapoptotic herpes simplex virus glycoprotein J localizes to multiple cellular organelles and induces reactive oxygen species formation. J. Virol..

[42] Wang J., Shen Y.H., Utama B., Wang J., LeMaire S.A., Coselli J.S., Vercellotti G.M., Wang X.L. (2006). HCMV infection attenuates hydrogen peroxide induced endothelial apoptosis-involvement of ERK pathway. FEBS Lett..

[43] Tilton C., Clippinger A.J., Maguire T., Alwine J.C. (2011). Human cytomegalovirus induces multiple means to combat reactive oxygen species. J. Virol..

[44] Sinclair J., Sissons P. (2006). Latency and reactivation of human cytomegalovirus. J. Gen. Virol..

[45] Korbecki J., Baranowska-Bosiacka I., Gutowska I., Chlubek D. (2013). The effect of reactive oxygen species on the synthesis of prostanoids from arachidonic acid. J. Physiol. Pharmacol..

[46] Chen K., Vita J.A., Berk B.C., Keaney J.F. (2001). c-Jun N-terminal kinase activation by hydrogen peroxide in endothelial cells involves SRC-dependent epidermal growth factor receptor transactivation. J. Biol. Chem..

